# Isolation, Identification, and Antimicrobial Activity of Secondary Metabolites from *Pseudophaeolus soloniensis* Against Phytopathogenic Fungi

**DOI:** 10.3390/jof12080601

**Published:** 2026-08-12

**Authors:** Chang Liu, Xiaoxue Zhao, Heng Zhao, Jun Zuo, Ruihan Wang, Mengshi Wang, Tianqi Wang, Jingyu Huang, Bin Du, Kui Niu

**Affiliations:** 1 College of Chemical Engineering, Hebei Normal University of Science and Technology, Qinhuangdao 066004, China; zxx4297@126.com (X.Z.); 18230248602@163.com (H.Z.); 15350674431@163.com (J.Z.); ruihwang@163.com (R.W.); 18232665658@163.com (T.W.); hzjy3657201@163.com (J.H.); 2 Hebei Key Laboratory of Natural Products Activity Components and Function, Hebei Normal University of Science and Technology, Qinhuangdao 066004, China; wms15233551083@163.com (M.W.); bindufood@aliyun.com (B.D.); 3 Engineering Research Centre of Chestnut Industry Technology Ministry of Education, Hebei Normal University of Science and Technology, Qinhuangdao 066004, China

**Keywords:** *Pseudophaeolus soloniensis*, antifungal activity, phytopathogenic fungi, solid-state fermentation, secondary metabolites, LC-MS, biocontrol

## Abstract

Plant pathogenic fungi pose a major threat to global crop production and food security, necessitating the development of sustainable control agents. This study investigated the antifungal potential of *Pseudophaeolus soloniensis*, a wood-decaying fungus. A wild strain was isolated from Hebei, China, and identified via morphology and ITS sequencing. A bioactive crude extract (P1) was obtained through optimized solid-state fermentation on A3M medium. Its antimicrobial spectrum was evaluated against major phytopathogenic fungi (e.g., *Fusarium graminearum*, *Aspergillus flavus*) and model bacteria (*Staphylococcus aureus*, *Escherichia coli*) using mycelial growth inhibition, Minimum Inhibitory Concentration (MIC), and agar diffusion assays. P1 exhibited strong, selective activity, showing significantly greater inhibition against *S. aureus* than *E. coli* and pronounced effects against *F. graminearum* (43.79% inhibition at 20 µg/mL) and *A. flavus* (73.5% at 0.2 mg/mL). A hormetic-like response was observed for *F. oxysporum*. Liquid Chromatography-Mass Spectrometry (LC-MS) analysis revealed a diverse secondary metabolome, including flavonoids, alkaloids, quinones, and saponins. These results establish *P. soloniensis* as a promising source of bioactive metabolites for developing eco-friendly fungicides.

## 1. Introduction

Plant diseases pose a significant threat to global agricultural productivity, accounting for 10–30% of annual crop losses worldwide [1]. Among the most damaging agents are pathogenic fungi, including field pathogens such as *Fusarium graminearum* and *Rhizoctonia solani*, which cause severe yield reductions [2], and storage fungi like *Aspergillus flavus*, which lead to post-harvest spoilage and contamination with hazardous mycotoxins [3,4].

*Fusarium graminearum*, the causal agent of Fusarium head blight (FHB), is a globally distributed pathogen that poses a significant threat to global food security by reducing crop yields and grain quality in major cereal crops, particularly wheat, barley, and maize [5]. *Aspergillus flavus* is a predominant post-harvest pathogen that contaminates major crops, including maize, peanuts, cottonseed, and tree nuts [4]. Maize is one of the most mycotoxin-exposed crops; except for patulin, all other major mycotoxins, including aflatoxins (AFB1), ochratoxin A (OTA), deoxynivalenol (DON), zearalenone (ZEN), and fumonisins (FUMs), occur at significant levels [3]. Multi-toxin contamination represents a serious food safety challenge, and despite growing research efforts, no significant decrease in mycotoxin contamination has been observed in the last decade [3]. Furthermore, climate warming—particularly warmer and hotter growing seasons—is expected to increase the risk of contamination by fumonisins and aflatoxins, exacerbating the economic and health impacts of these pathogens [3].

The predominant reliance on chemical fungicides for disease management has led to serious problems, including environmental pollution, the evolution of pathogen resistance, and concerns over chemical residues in food [5,6]. This situation underscores the critical need for the development of sustainable and eco-friendly alternatives for crop protection.

Fungi are prolific producers of diverse secondary metabolites with a broad spectrum of bioactivities [7]. These compounds, which include structural classes such as terpenoids, alkaloids, and polyketides [8,9], have been successfully exploited in medicine, as exemplified by antibiotics like penicillin [10]. Their often-novel mechanisms of action also make them promising candidates for overcoming pesticide resistance [11]. In agriculture, bioactive metabolites from fungi, particularly wood-decaying basidiomycetes in the Polyporaceae family, offer a valuable resource for developing new biopesticides [12,13].

*Pseudophaeolus soloniensis* (formerly *Piptoporellus soloniensis*) is a wood-decaying Polyporaceae fungus known to produce a variety of secondary metabolites in its fruiting bodies and mycelia [14]. Previous research on this species has primarily focused on taxonomic classification [15,16] and the isolation of bioactive compounds with diverse activities. Recent studies have reported the isolation of compounds with anti-tumor and anti-inflammatory activities [17,18], as well as antimicrobial properties [19]. Most notably, a recent study by Guo et al. [20] isolated three novel aromatic meroterpenoids (soloniols A–C) from *P. soloniensis* mycelia, which demonstrated significant antifungal activity against phytopathogenic fungi, including *Rhizoctonia solani*, *Fusarium avenaceum*, and *Fusarium asiaticum*. Specifically, soloniol A exhibited inhibition rates of 40.7–59.8% at 200 μg/mL against these pathogens [20]. This finding is particularly relevant as *F. asiaticum* is a member of the *F. graminearum* species complex, and *R. solani* is also evaluated in our study.

Despite this emerging evidence of bioactivity, the antimicrobial potential of *P. soloniensis* remains largely unexplored from an agricultural biocontrol perspective. A systematic evaluation of its activity against a comprehensive panel of economically important plant pathogens—particularly major mycotoxin producers such as *F. graminearum* and *A. flavus*—has not been conducted. Furthermore, the relationship between crude extract activity and specific metabolite classes under different fermentation conditions requires investigation.

To address these gaps, the present study aimed to: (1) isolate and identify a wild strain of *P. soloniensis* from Hebei Province, China, and optimize culture conditions using solid-state fermentation; (2) systematically evaluate the antimicrobial activity of the resulting crude extract against major phytopathogenic fungi (including *F. graminearum*, *A. flavus*, and *R. solani*) and model bacteria; and (3) profile its secondary metabolites using LC-MS to identify putative bioactive constituents and compare with reported compounds. The findings confirm the potential of *P. soloniensis* as a promising reservoir of antifungal metabolites for sustainable agriculture.

## 2. Materials and Methods

### 2.1. Fungal Material, Isolation, and Identification

Fruiting bodies of *Pseudophaeolus soloniensis* were collected from a forest in Lulong County, Hebei Province, China. A pure culture was obtained from tissue samples and maintained on potato dextrose agar (PDA) slants (Beijing Aoboxing Bio-Tech Co., Ltd., Beijing, China). For long-term storage, slants fully covered with mycelium were kept at 4 °C. Morphological identification was based on macroscopic features of the colony, including color, texture, margin, and aerial mycelium. For molecular identification, genomic DNA was extracted from fresh mycelia using a modified cetyltrimethylammonium bromide (CTAB) method [21] with RNase (Sigma-Aldrich, St. Louis, MO, USA). The internal transcribed spacer (ITS) region of ribosomal DNA was amplified by polymerase chain reaction (PCR) using an electrophoresis apparatus (Model DYY-7B, Beijing Liuyi Instrument Factory, Beijing, China) with the universal primers ITS1F and ITS4 [22,23]. The purified PCR products were bidirectionally sequenced by Shanghai Parsley Biotechnology Co., Ltd. (Shanghai, China). The obtained sequences were compared to reference sequences in the NCBI GenBank database using the BLASTn algorithm. A phylogenetic tree was constructed with the neighbor-joining method in MEGA software (version 7.0). The newly determined ITS sequence was deposited in GenBank under accession number PQ870053.

### 2.2. Fermentation Optimization and Crude Extract Preparation

To select an optimal growth medium, preliminary screening was conducted on three solid or semi-solid media: A3M, A11M, and A16 (compositions listed in Supplementary Table S1). *P. soloniensis* exhibited vigorous growth only on A3M. Consequently, large-scale solid-state fermentation was performed on A3M medium in Petri dishes (NEST Biotechnology Co., Ltd., Wuxi, China). Cultures were incubated at 28 °C in the dark for 35 days in a biochemical incubator (Model SPX-250B, Jintan Hongkai Instrument Factory, Changzhou, China). The fermented solid substrate was extracted three times by ultrasonication (200 W, 30 min each) using an *n*-butanol/water (1:1, *v*/*v*) mixture (Tianjin Oubokai Chemical Co., Ltd., Tianjin, China). The combined organic phases were concentrated under reduced pressure at 40 °C using a rotary evaporator (Model N-1100, Tokyo Rikakikai Co., Ltd., Tokyo, Japan), yielding a brown, viscous crude extract designated as P1. The extract was weighed on an electronic balance (Model LT302B, Tianjin Oubokai Chemical Product Sales Co., Ltd., Tianjin, China) (1.4 g from 5 L fermentation culture), stored at 4 °C in a refrigerator (Model BCD-555WKPZM, Midea Electric Co., Ltd., Hefei, China), and protected from light until use. This single batch of extract (designated P1) was used for all subsequent antimicrobial assays and LC-MS analysis to ensure experimental consistency.

### 2.3. Evaluation of Antimicrobial Activity of P1

#### 2.3.1. Determination of Bacterial Minimum Inhibitory Concentration (MIC)

The MIC of P1 against *Staphylococcus aureus* (ATCC 25923) and *Escherichia coli* (ATCC 25922) was determined using the broth microdilution method according to the Clinical and Laboratory Standards Institute (CLSI) guidelines with modifications. Briefly, bacterial suspensions were adjusted to a density of 1 × 10^6^ CFU/mL in LB broth. In a 96-well microtiter plate, P1 was serially diluted two-fold in LB broth across a concentration range (2 to 0.0002 mg/mL). An equal volume of bacterial inoculum was added to each well. Wells containing only inoculum (negative control) and only medium with P1 (blank control) were included. The plate was incubated at 37 °C for 18 h. Bacterial growth was assessed by measuring the optical density at 600 nm (OD_600_) using a microplate reader (Multiskan GO, Thermo Fisher Scientific, Vantaa, Finland). The percentage inhibition was calculated as follows:

$$\mathrm{Inhibition}\;(\%)=\left[1-\frac{\left(OD_{\mathrm{treatment}}\;-\;OD_{\mathrm{blank}}\right)}{\left(OD_{\mathrm{control}}\;-\;OD_{\mathrm{blank}}\right)}\right]\times 100\%$$

where $OD_{\mathrm{treatment}}$, $OD_{\mathrm{control}}$, and $OD_{\mathrm{blank}}$ represent the optical densities of the sample well, the negative control well, and the blank control well, respectively. The MIC was defined as the lowest concentration that inhibited ≥90% of visible growth. All assays were performed in quadruplicate.

#### 2.3.2. Agar Well Diffusion Assay

The agar well diffusion method was used to further assess antibacterial and antifungal activity [24]. For bacteria, suspensions (1 × 10^8^ CFU/mL) were spread onto LB agar plates. For fungi (*Aspergillus flavus* and *A. niger*), spore suspensions (1 × 10^6^ spores/mL) were mixed with molten PDA before solidification. Wells (6 mm diameter) were punched into the agar and filled with 60 µL of P1 at various concentrations (50, 20, 10, 2, and 0.2 mg/mL). Sterile saline served as the negative control. After incubation (37 °C for 24 h for bacteria; 28 °C for 48–72 h for fungi), the diameters of the inhibition zones (including the well) were measured. Experiments were conducted with four replicates.

#### 2.3.3. Mycelial Growth Inhibition Assay

The antifungal activity of P1 against filamentous phytopathogens was evaluated using the mycelial growth inhibition method on PDA plates [25,26]. Two experimental setups were used due to differing pathogen growth rates and sensitivity ranges.

For *Fusarium* spp. and *Rhizoctonia solani*: A stock solution of P1 in dimethyl sulfoxide (DMSO, Sigma-Aldrich, St. Louis, MO, USA) was incorporated into PDA to achieve final concentrations of 20 and 80 µg/mL. A mycelial plug (5 mm diameter) from a 7-day-old culture was placed at the center of each plate. Plates containing an equivalent amount of DMSO and plates containing carbendazim (Aladdin, Shanghai, China) (100 mg/L) served as the negative and positive controls, respectively. Plates were incubated at 28 °C in the dark.

For *Aspergillus* spp., *Mucor* sp., and *Rhizopus* sp.: P1 was directly added to cooled, molten PDA to final concentrations of 0.2, 0.02, 0.002, and 0.0002 mg/mL before pouring plates. Mycelial plugs (4 mm diameter) from 7-day-old cultures were used as inocula. Sterile saline and carbendazim (0.02, 0.002, 0.0002 mg/mL) served as negative and positive controls, respectively. Plates were incubated at 25 °C in the dark.

For both setups, when the mycelium in the negative control plate covered approximately 80% of the surface, the colony diameter in each treatment was measured along two perpendicular axes. The mycelial growth inhibition rate was calculated using the formula:

$$\mathrm{Inhibition}\;(\%)=\frac{\left(D_{c}\;-{\;D}_{t}\right)}{\left(D_{c\;}-\;d\right)}\times 100\%$$

where $D_{c}$ is the average colony diameter in the negative control, $D_{t}$ is the average colony diameter in the treatment, and d is the diameter of the inoculum plug (5 mm or 4 mm). All experiments were performed with four replicates, and results are presented as mean ± standard deviation (SD).

### 2.4. LC-MS Analysis of Secondary Metabolites

The chemical profile of crude extract P1 was analyzed using liquid chromatography coupled with quadrupole time-of-flight mass spectrometry (LC-QTOF-MS). An Agilent 6545 Q-TOF system (Agilent Technologies Inc., Santa Clara, CA, USA) equipped with an Agilent ZORBAX Eclipse Plus C18 column (50 mm × 2.1 mm, 1.8 µm particle size, Agilent Technologies Inc., Santa Clara, CA, USA) was used. Prior to injection, P1 was dissolved in 100% methanol, filtered through a 0.22 μm membrane, and a 3 μL aliquot was injected. Chromatographic separation was achieved at a flow rate of 0.45 mL/min using a binary gradient of (A) 0.1% formic acid in water and (B) acetonitrile. The gradient program was: 5% B (0–2 min), 5–100% B (2–6 min), 100% B (6–8 min), 100–5% B (8–8.5 min), and 5% B (8.5–10 min). The mass spectrometer was operated in positive electrospray ionization (ESI+) mode with a data-dependent acquisition (DDA) method. Key source parameters were: spray voltage, 3.5 kV; capillary temperature, 250 °C; and sheath gas temperature, 300 °C.

## 3. Results

### 3.1. Morphological and Molecular Identification of Pseudophaeolus soloniensis

**Figure 1:**
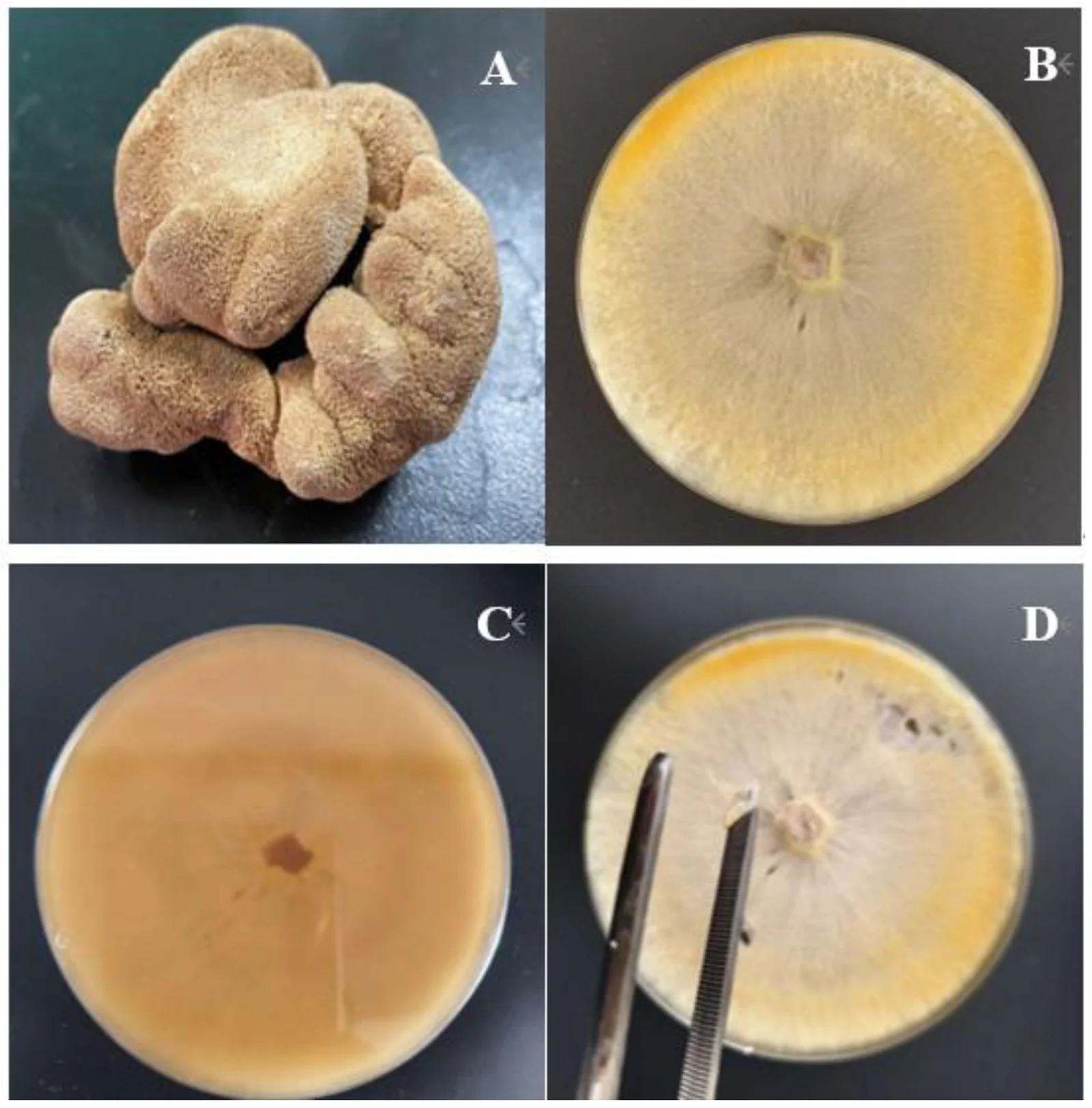
*Pseudophaeolus soloniensis*. (**A**) Wild fruiting bodies. (**B**) Obverse of colony. (**C**) Reverse side of colony. (**D**) Mycelia.

A strain was successfully isolated from the fruiting bodies of *P. soloniensis* collected in Hebei Province, China (Figure 1A). After 7 days of cultivation on PDA medium, colonies exhibited a milky-white color, a moderately felt-like texture, and a growth rate reaching 70–90 mm in diameter. Colony margins were typically undulate, with abundant aerial mycelium development (Figure 1B–D) [27].

**Figure 2:**
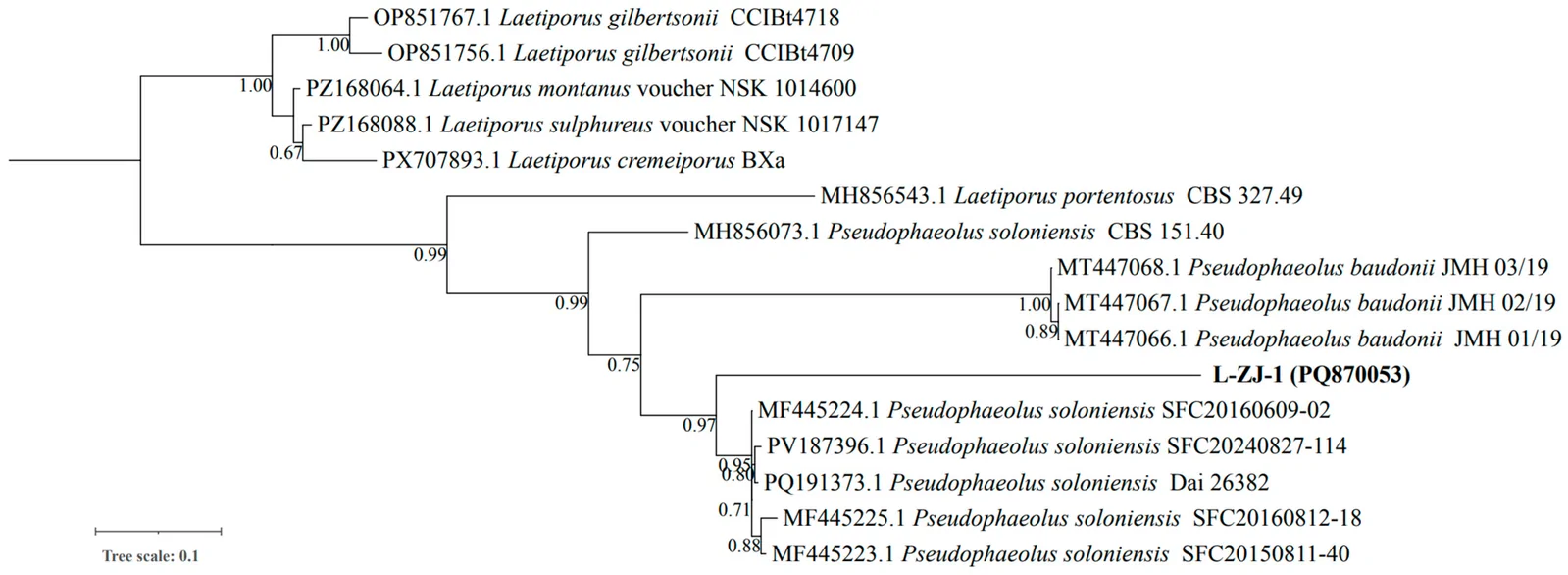
Phylogenetic tree of *Piptoporellus soloniensis* inferred from ITS sequences using the neighbor-joining method. Bootstrap values (≥0.67, based on 1000 replicates) are shown at the nodes. The scale bar (0.1) represents the evolutionary distance corresponding to 0.1 nucleotide substitutions per site (i.e., 10% sequence divergence). The strain isolated in this study is marked with a black box and designated as L-ZJ-1 (PQ870053).

To confirm precise taxonomic placement, molecular identification was performed via sequencing of the internal transcribed spacer (ITS) region of ribosomal DNA. BLASTn analysis revealed that the obtained ITS sequence showed a high similarity (≥98.7%) with the reference sequence of *Pseudophaeolus soloniensis* deposited in the NCBI GenBank database. Phylogenetic analysis using the neighbor-joining method further confirmed its classification within the *P. soloniensis* clade with strong bootstrap support (Figure 2). It is noteworthy that *Piptoporellus soloniensis* has been reclassified as *Pseudophaeolus soloniensis* based on recent phylogenetic studies (NCBI Taxonomy ID: 270283). In this paper, we adopt the currently accepted name, *Pseudophaeolus soloniensis*, to ensure taxonomic consistency.

### 3.2. Optimization of Solid-State Fermentation for Biomass and Metabolite Production

Fungi have the ability to produce secondary metabolites based on substrate and environmental factors. The existence of a suitable medium for generating bioactive secondary metabolites is of great significance as it supports the production of metabolites. Preliminary growth screening was performed on three solid or semi-solid media (A3M, A11M, and A16; compositions provided in Supplementary Table S1). The *P. soloniensis* isolate exhibited vigorous mycelial growth exclusively on A3M solid medium after 7 days of incubation at 30 °C, whereas growth on the other tested media was negligible. This emphasizes the significant influence of both medium composition and physical factors on the growth of filamentous fungi [28]. Based on this preference, A3M solid medium was selected for scaled-up solid-state fermentation (SSF). After 35 days of SSF, the culture was extracted using a n-butanol/water system, and 1.4 g of a brown crude extract (designated P1) was extracted from 5 L of fermentation products. The successful implementation of SSF for this species provides a practical framework for generating sufficient material for downstream chemical and biological analyses.

### 3.3. Antimicrobial Activity of Crude Extract P1

#### 3.3.1. Antibacterial Activity Against *S. aureus* and *E. coli*

**Figure 3:**
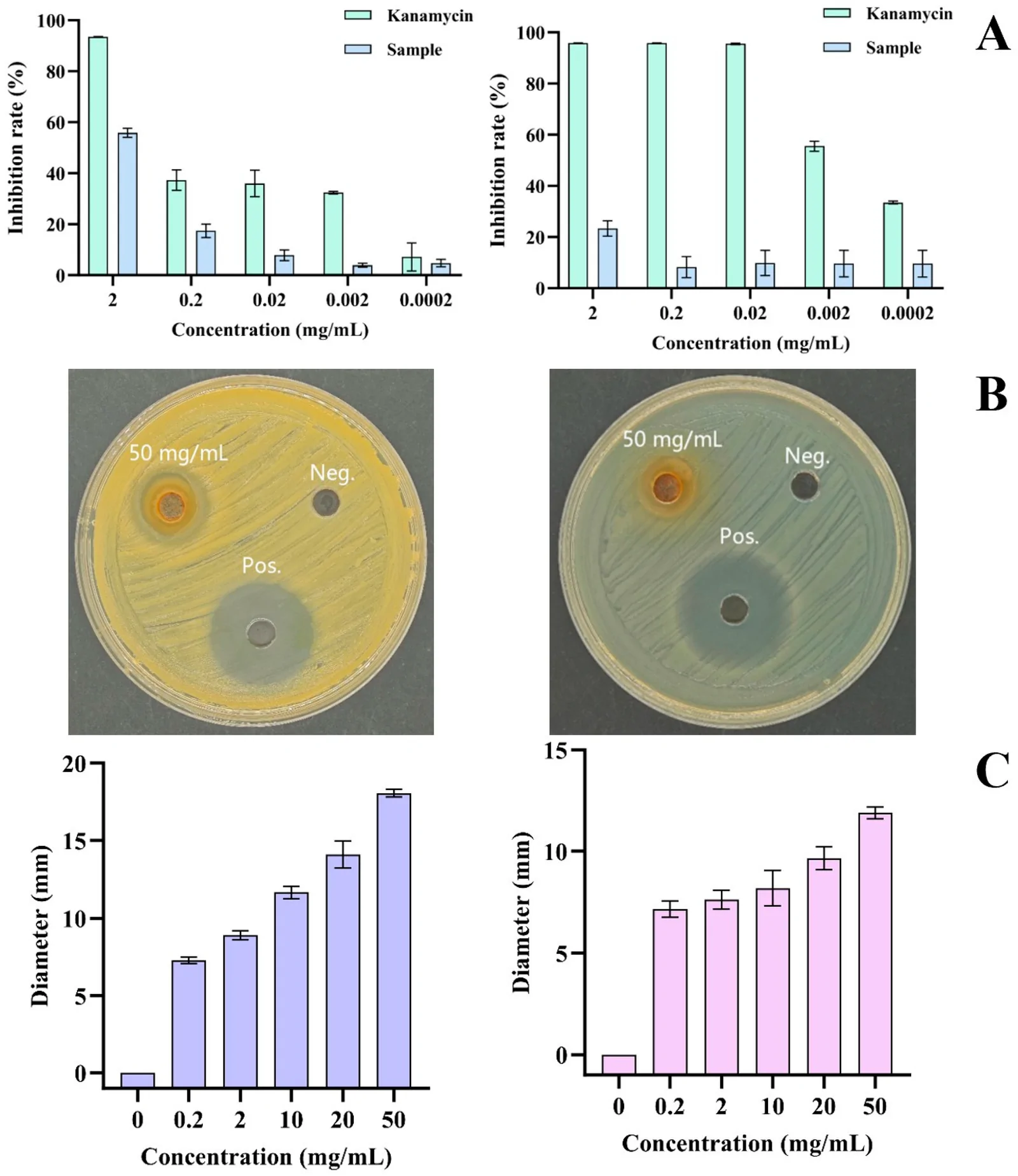
Antibacterial activity of crude extract P1 against *Staphylococcus aureus* and *Escherichia coli* (left: *S. aureus*; right: *E. coli*). (**A**) Minimum Inhibitory Concentration (MIC) assay results. (**B**) Representative inhibition-zone photographs at 50 mg/mL. (**C**) Quantitative comparison of inhibition-zone diameters. Kanamycin and sterile saline served as positive and negative controls, respectively.

The antibacterial activity of P1 was evaluated against Gram-positive (*S. aureus*) and Gram-negative (*E. coli*) model strains using MIC and inhibition zone assays. In the MIC assay, P1 exhibited a concentration-dependent bactericidal effect against *S. aureus*, with activity increasing progressively across the tested range (0.0002–2 mg/mL); however, its potency was markedly lower than that of kanamycin (Figure 3A), consistent with the nature of a crude extract wherein active constituents are present in an unpurified matrix [29]. Notably, P1 was virtually inactive against *E. coli* at all concentrations examined, revealing a pronounced Gram-positive selective inhibition profile. Corroborating these findings, the inhibition zone assay demonstrated a clear concentration-dependent response against *S. aureus*, with a maximum zone of 18.07 ± 0.25 mm at 50 mg/mL, which diminished progressively to 12.50 ± 0.18 mm at 10 mg/mL and became negligible at 0.2 mg/mL (Figure 3C). Representative photographs of inhibition zones at 50 mg/mL are presented in Figure 3B. For *E. coli*, only a marginal zone of 11.90 ± 0.30 mm was observed at 50 mg/mL, with no measurable inhibition at lower concentrations, further confirming the selective activity of P1.

#### 3.3.2. Antifungal Activity Against *Fusarium* spp. and *Rhizoctonia solani*

**Figure 4:**
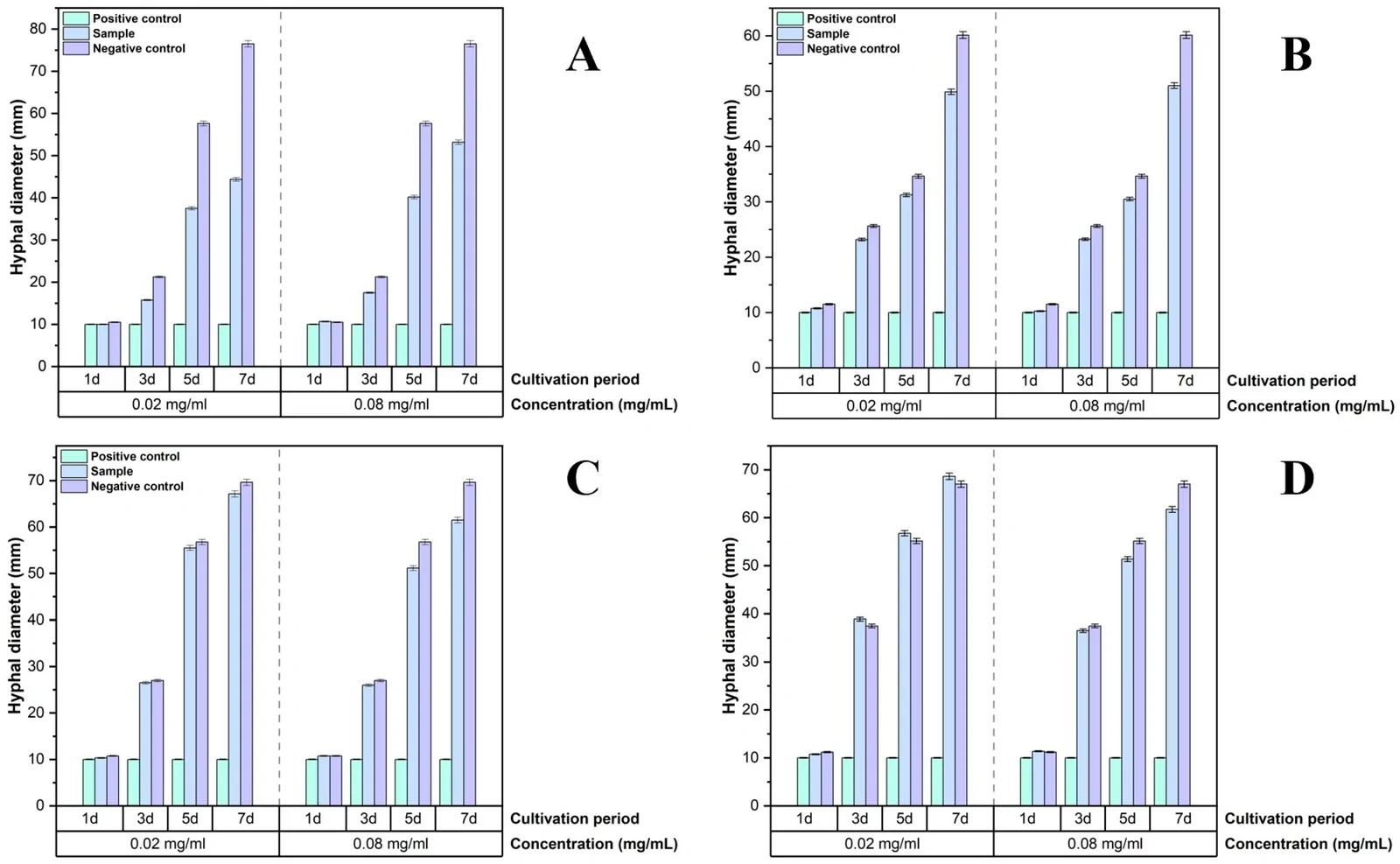
Antifungal activity of crude extract P1 against *Fusarium graminearum*, *F. verticillioides*, *Rhizoctonia solani*, and *Fusarium oxysporum*. (**A**) *F. graminearum*. (**B**) *F. verticillioides*. (**C**) *R. solani*. (**D**) *F. oxysporum*. Carbendazim (100 mg/L) served as the positive control, and DMSO as the negative control.

The crude extract was analyzed for its antifungal activity against *Fusarium graminearum*, *Fusarium verticillioides*, *Rhizoctonia solani* and *Fusarium oxysporum*. The results demonstrated a clear and selective antifungal profile (Figure 4A–D).

The most significant inhibitory activity was observed against *F. graminearum*, which is the main pathogenic fungus causing Fusarium head blight of grains. At a concentration of 20 µg/mL, P1 exhibited time-dependent inhibition, reaching a maximum of 43.79% on day 7. Notably, a higher concentration of 80 µg/mL resulted in a lower inhibition rate (27.94% on day 7) (Figure 4A). In contrast, P1 exhibited limited activity against *F. verticillioides* and *Rhizoctonia solani*. Even at 80 µg/mL, the maximum inhibition rates were only 11.91% and 11.67%, respectively (Figure 4B,C). A notable hormetic response was observed in *F. oxysporum*: at 20 µg/mL, mild growth stimulation (−3.67% inhibition) was recorded, whereas 80 µg/mL produced only 7.84% inhibition (Figure 4D).

#### 3.3.3. Antifungal Activity Against *Aspergillus* spp., *Mucor* sp., and *Rhizopus* sp.

**Figure 5:**
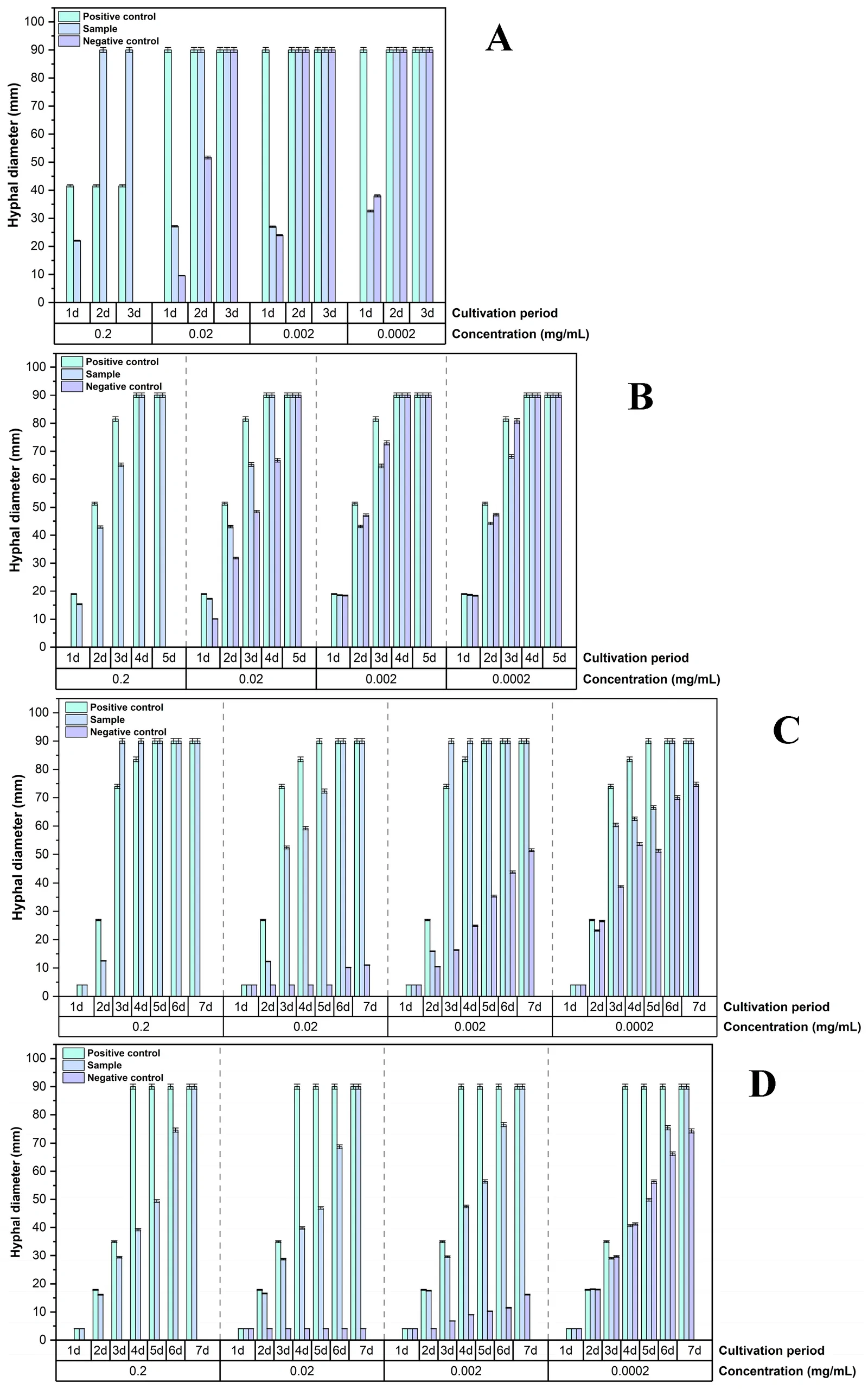
Antifungal activity of crude extract P1 against *Rhizopus* sp., *Mucor* sp., *Aspergillus niger*, and *A. flavus*. (**A**) *Rhizopus* sp. (**B**) *Mucor* sp. (**C**) *A. niger*. (**D**) *A. flavus*. Carbendazim served as the positive control, and sterile saline as the negative control.

The crude extract was analyzed for its antifungal activity against *Aspergillus flavus*, *Aspergillus niger*, *Mucor* sp., and *Rhizopus* sp. The results demonstrated a clear and selective antifungal profile (Figure 5A–D).

As shown in Figure 5A,B, P1 exerted only limited, transient inhibition against *Mucor* sp. and *Rhizopus* sp. At 0.2 mg/mL, inhibition rates reached 24.0% and 38.5% on day 1, respectively, but rapidly diminished thereafter, with negligible effects by day 4. In contrast, *Aspergillus* spp. proved considerably more susceptible. At 0.2 mg/mL, P1 inhibited *A. niger* mycelial growth by 72.6% on day 3, and colony diameters remained substantially smaller than the control through day 7 (Figure 5C). Against *A. flavus*, the inhibitory effect of P1 was both dose and time dependent. As depicted in Figure 5D, at 0.2 mg/mL, inhibition increased from 59.0% (day 4) to 63.8% (day 5) and reached 73.5% by day 6. Even at a tenfold lower concentration (0.02 mg/mL), P1 still suppressed *A. flavus* growth by 47.3% on day 5.

### 3.4. Analysis of Secondary Metabolites by LC-MS

**Figure 6:**
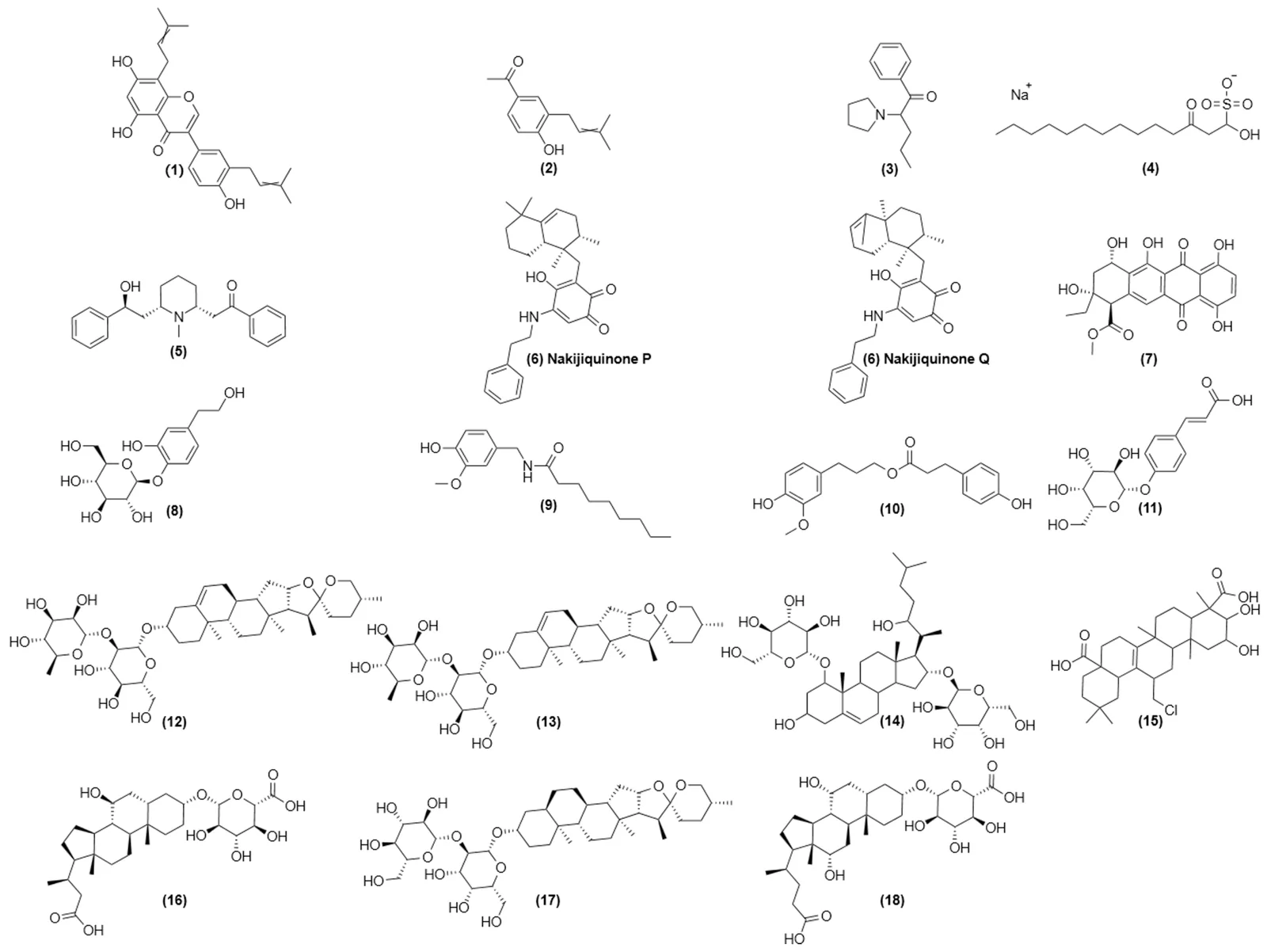
Chemical structures of the 19 major secondary metabolites (including Peaks **1**–**18**, with Peak **6** representing both Nakijiquinone P and Q) tentatively identified in the crude extract P1 of *Pseudophaeolus soloniensis* via LC-MS profiling. (Detailed compound names, molecular formulas, and mass errors are provided in Table 1).

The integrated Agilent 6545 Q-TOF LC/MS system was used to characterize the secondary metabolites in crude extract P1. Based on exact mass, isotope patterns, and comparisons with spectral databases or literature data, a total of 18 major peaks (representing 19 compounds) were detected and preliminarily identified (Table 1). The metabolites include flavonoids, phenylpropanoids, alkaloids, quinones, terpenoids, and saponins, highlighting the chemical richness of *P. soloniensis* under the applied solid-state fermentation conditions. Among these, putative rutilantinone (peak **7**, *m*/*z* 451.0995, RT 8.195 min) was tentatively identified as a dominant quinone component, while putative nonivamide (peak **9**, *m*/*z* 316.1830, RT 6.151 min) and putative timosaponin A3 (peak **17**, *m*/*z* 763.4204, RT 6.965 min) represented the major capsaicinoid and steroidal saponin constituents, respectively. Additional peaks corresponding to flavonoids, alkaloids, and other phenolic derivatives were also tentatively detected (Table 1), with their respective chemical structures illustrated in Figure 6.

## 4. Discussion

The growing incidence of fungicide resistance among phytopathogenic fungi, coupled with increasing public concern over chemical residues in food and the environment, has intensified the search for sustainable alternatives to conventional crop protection agents. Wood-decaying basidiomycetes of the Polyporaceae family have emerged as particularly promising reservoirs of structurally diverse secondary metabolites endowed with antimicrobial properties [12]. It has been estimated that approximately 75% of polypore fungi that have been tested exhibit strong antimicrobial activity, rendering them a valuable resource for the development of novel biocontrol agents [30]. The present study provides the first comprehensive evaluation of *Pseudophaeolus soloniensis* as a reservoir of bioactive metabolites with activity against economically important phytopathogenic fungi and a detailed chemical profiling of its secondary metabolome under solid-state fermentation conditions.

While our species identification was based primarily on ITS sequencing combined with morphological characteristics—the standard approach in fungal taxonomy—we acknowledge that multi-locus phylogenetic analysis using additional markers (e.g., LSU, TEF1-α) could provide enhanced phylogenetic resolution. Nevertheless, the high ITS sequence similarity (≥98.7%) and morphological concordance provide robust evidence for the identification of our isolate as *P. soloniensis*. The selection of A3M solid medium as the production matrix was guided by preliminary growth screening that revealed a clear preference of *P. soloniensis* for this formulation. Solid-state fermentation (SSF) more closely mimics the natural growth conditions of wood-decaying fungi, and evidence indicates that the low water activity characteristic of solid substrates can influence fungal growth, gene expression, and the secretion of extracellular enzymes and other secondary metabolites [31,32]. This physiological adaptation likely contributed to the successful recovery of 1.4 g of crude extract from 5 L of fermented material in the present study.

The crude extract P1 exhibited a pronounced Gram-positive selective antibacterial profile, with substantial activity against *S. aureus* but negligible activity against *E. coli* across all tested concentrations. This differential susceptibility is consistent with the well-documented permeability barrier conferred by the Gram-negative outer membrane, which comprises a rigid leaflet of lipopolysaccharide with narrow porins that restrict the penetration of hydrophilic solutes and amphiphilic molecules [33]. Recent studies have similarly reported that Gram-positive bacteria are generally more sensitive than Gram-negative bacteria to the antibacterial effects of botanical and fungal extracts [34]. From an agricultural biocontrol perspective, the observed selectivity is advantageous, as it implies that P1 could target Gram-positive phytopathogens while exerting minimal collateral impact on the Gram-negative soil microbiota that are integral to nutrient cycling and plant health.

Among the nine phytopathogenic fungi tested, the crude extract P1 exhibited the most notable activity against *F. graminearum* (43.79% inhibition at 20 µg/mL) and *A. flavus* (73.5% inhibition at 0.2 mg/mL). Notably, the observed time-dependent accumulation of inhibitory activity against *A. flavus* is consistent with the documented mode of action of membrane-targeting metabolites, which impair membrane integrity and induce oxidative stress [35]. This finding carries practical significance, given that *A. flavus* is a major toxin-producing post-harvest pathogen responsible for substantial economic losses and serious public health risks worldwide [36]. In contrast, the limited and transient activity observed against *Rhizopus* sp. and *Mucor* sp. aligns with the well-documented intrinsic resistance of Mucorales to multiple antifungal agents [37]. Mucorales possess distinctive cell wall architecture, and efflux pumps conferring resistance have been widely documented in this order, contributing to their broad resistance profile [38].

The differential activity of P1 among the Fusarium species tested warrants careful consideration. Pronounced activity against *F. graminearum* contrasted sharply with the limited efficacy against *F. verticillioides* and *F. oxysporum*, and a hormetic-like growth stimulation was observed in the latter at 20 µg/mL. Hormesis, defined as a biphasic dose–response in which low doses stimulate growth while high doses inhibit it, has been documented for a range of filamentous fungi, including *Sclerotinia sclerotiorum*, upon exposure to sub-inhibitory concentrations of fungicides [39,40]. The underlying mechanisms are thought to involve the activation of stress-adaptive pathways, such as the upregulation of efflux pumps and detoxification enzymes, that can transiently enhance fungal metabolic activity and growth at low xenobiotic concentrations [41]. The finding that hormesis occurred in *F. oxysporum* but not in *F. graminearum* or *F. verticillioides* highlights the species-specific nature of fungal stress responses and underscores the need for tailored application strategies when using crude natural product extracts.

It is noteworthy that specific aromatic meroterpenoids (soloniols A–C) recently reported by Guo et al. [20] were not detected in our LC-MS analysis. However, we tentatively identified several putative prenylated aromatic compounds characterized by 3-methylbut-2-enyl (prenyl) substituents attached to flavonoid, phenolic, and quinone cores (Table 1, peaks **1**, **2**, and **7**). While these compounds are not classical meroterpenoids in the strict biosynthetic sense, the presence of prenyl groups indicates active isoprenoid biosynthetic machinery (MVA or MEP pathways) and prenyltransferase activity in our *P. soloniensis* strain. These prenylated compounds may represent biosynthetic intermediates or alternative products of the same gene clusters that produce true meroterpenoids under different conditions.

The absence of soloniols may be attributed to: (1) differences in fungal material—Guo et al. [20] extracted from mycelia cultured in liquid medium, whereas we used solid-state fermentation on A3M medium, which can activate different biosynthetic gene clusters; (2) variations in extraction methodology—we used n-butanol/water (1:1, *v*/*v*), while different solvent polarities may selectively extract different meroterpenoid classes; (3) strain-specific biosynthetic variation between our Hebei isolate and strains from other geographical origins; and (4) fermentation duration and environmental conditions (35 days at 28 °C in darkness in our study vs. different conditions in previous reports). Interestingly, Guo et al. reported that soloniol A exhibited 40.7–59.8% inhibition against *F. asiaticum* at 200 μg/mL [20], whereas our crude extract P1 achieved similar inhibition (43.79%) at a 10-fold lower concentration (20 μg/mL). This enhanced potency per unit mass, despite the absence of detectable soloniols in our LC-MS analysis, suggests one of the following scenarios: (1) our extract may contain structurally related putativeprenylated compounds (peaks **1**, **2**, **7**) with comparable or superior antifungal activity; (2) synergistic interactions among multiple bioactive constituents (e.g., saponins, quinones, prenylated aromatics) may enhance overall efficacy; or (3) differences in extraction efficiency may have resulted in higher relative concentrations of bioactive non-meroterpenoid compounds. This observation underscores the biosynthetic plasticity of *P. soloniensis* and warrants further investigation through bioassay-guided fractionation.

This observation underscores the profound influence of cultivation parameters on the fungal secondary metabolome [31,32] and highlights the biosynthetic plasticity of *P. soloniensis*. Future studies employing OSMAC (One Strain Many Compounds) approaches—systematic variation in culture conditions, media composition, and extraction methods—could potentially unlock additional meroterpenoid diversity and reveal the full biosynthetic potential of this species.

The LC-MS profiling of P1 provided a chemical rationale for the observed antimicrobial activities. Among the tentatively identified compounds, several are documented to possess antimicrobial properties. Putative nonivamide (peak **9**), a capsaicin analogue, is suggested to exert antimicrobial activity primarily through disruption of cell membrane integrity and inhibition of bacterial efflux pumps [42,43], suggesting a potential role in the Gram-positive selective antibacterial activity of P1. A series of putative steroidal glycosides and saponins (peaks **13**, **14**, **17**), including putative timosaponin A3 (peak **17**), were also tentatively identified; these amphiphilic molecules are proposed to interact with ergosterol in fungal membranes, increasing permeability and causing leakage of cytoplasmic contents—a well-established antifungal mechanism [44]. Of particular note, putative rutilantinone (peak **7**), a class I anthracycline-type quinone, was tentatively detected. Current evidence, though limited, indicates its activity against Gram-positive bacteria (including MRSA) with low acute toxicity and a low tendency to induce resistance [45], and suggests a mechanism involving interference with macromolecular synthesis [46]. This metabolite profile rationalizes the observed antimicrobial spectrum of P1 and provides a chemical foundation for subsequent network pharmacology analysis and bioassay-guided fractionation.

Several limitations of the present study should be acknowledged. First, while the crude extract P1 was tested across a broad concentration range against a comprehensive panel of target organisms, the observed bioactivity is likely attributable to a subset of the detected metabolites, and the relative contribution of individual compounds remains to be determined. Second, the antimicrobial assays were conducted exclusively under in vitro conditions; the efficacy of P1 or its purified components in planta remains unknown. Third, the hormetic response observed in *F. oxysporum* underscores the need for careful dose optimization and potentially tailored application strategies when using crude natural product extracts. Finally, the toxicity profile of P1 toward non-target organisms has not been evaluated. Addressing these limitations, particularly in vitro efficacy and toxicity, will be critical for future development.

## 5. Conclusions

This study provides the first comprehensive evaluation of *Pseudophaeolus soloniensis* as a biocontrol resource for agriculture. The isolation and molecular confirmation of the strain from Hebei Province extends the known geographical distribution of this species. Solid-state fermentation on A3M medium yielded a metabolically rich crude extract (P1) with a selective antimicrobial spectrum. P1 demonstrated pronounced inhibition against the field pathogen *Fusarium graminearum* and the storage fungus *Aspergillus flavus*, both economically critical targets, while displaying limited activity against Mucorales and a clear Gram-positive selective antibacterial profile. LC-MS profiling of P1 revealed a diverse array of secondary metabolites, including flavonoids, alkaloids, quinones, and steroidal and triterpenoid saponins, among which putative rutilantinone, nonivamide, and timosaponin A3 were tentatively identified as candidate bioactive constituents. These findings establish *P. soloniensis* as a previously overlooked reservoir of antifungal secondary metabolites for agricultural applications and provide a chemical and biological rationale for the potential development of targeted, eco-friendly biocontrol agents. Future investigations should focus on bioassay-guided isolation of active compounds, elucidation of their mechanisms of action, and assessment of field efficacy and environmental safety.

## Figures and Tables

**Table 1:** LC-MS (Liquid Chromatography-Mass Spectrometry peaks identify the major secondary metabolites obtained from crude extract P1.

Peak No.	Compound	Molecular Formula	Theoretical Exact Mass (*m*/*z*)	Experimental Exact Mass (*m*/*z*)	Mass Error (ppm)	RT (min)	Compound Class
**1** *	5,7-dihydroxy-3-[4-hydroxy-3-(3-methylbut-2-enyl)phenyl]-8-(3-methylbut-2-enyl)chromen-4-one	C_25_H_26_O_5_	407.1853 [M+H]^+^	407.1855	+0.5	4.655	Prenylated flavonoid
**2** *	1-[4-hydroxy-3-(3-methylbut-2-enyl)phenyl]ethanone	C_13_H_16_O_2_	205.1223 [M+H]^+^	205.1226	+1.4	6.035	Prenylated phenolic ketone
**3**	α-Pyrrolidinovalerophenone	C_15_H_21_NO	232.1696 [M+H]^+^	232.1644	−22.2	6.35	Alkaloid
**4**	Sodium houttuyfonate	C_14_H_27_O_5_S	308.1600 [M+H]^+^	308.1599	−0.3	7.996	Organosulfur compound
**5**	Lobeline hydrochloride	C_22_H_27_NO_2_	360.1934 [M+Na]^+^	360.1936	+0.6	8.611	Alkaloid
**6**	Nakijiquinone P/Q	C_29_H_37_NO_3_	448.2846 [M+H]^+^	448.2867	+4.6	5.154	Quinone alkaloid
**7** *	Rutilantinone	C_22_H_20_O_9_	451.0999 [M+Na]^+^	451.0995	−0.8	8.195	Prenylquinone
**8**	2-Hydroxy-4-(2-hydroxyethyl)phenyl β-D-glucopyranoside	C_14_H_20_O_8_	317.1231 [M+H]^+^	317.1203	−8.8	5.054	Phenolic glycoside
**9**	Nonivamide	C_17_H_27_NO_3_	316.1799 [M+Na]^+^	316.1830	+9.7	6.151	Capsaicinoid
**10**	3-(4-hydroxy-3-methoxyphenyl)propyl 3-(4-hydroxyphenyl)propanoate	C_19_H_22_O_5_	348.1805 [M+NH_4_]^+^	348.1787	−5.2	6.168	Phenolic ester
**11**	Phenylpropanoid derivative	C_15_H_18_O_8_	327.1075 [M+H]^+^	327.1063	−3.8	6.45	Phenylpropanoid
**12**	4-Acetoxyphenol	C_8_H_8_O_3_	175.0366 [M+Na]^+^	175.0342	−13.5	7.248	Phenolic ester
**13**	Prosapogenin A	C_39_H_62_O_12_	745.4100 [M+Na]^+^	745.4171	+9.5	5.121	Steroidal saponin
**14**	Alloside B	C_39_H_66_O_14_	759.4525 [M+H]^+^	759.4523	−0.2	5.154	Steroidal glycoside
**15**	Senegenin	C_30_H_45_ClO_6_	537.2900 [M+H]^+^	537.2909	+1.7	5.37	Triterpenoid saponin
**16**	(2*S*,3*S*,4*S*,5*R*,6*R*)-6-(((3*R*,5*R*,7*S*,8*R*,9*S*,10*S*,13*R*,14*S*,17*R*)-17-((*R*)-1-carboxypropan-2-yl)-7-hydroxy-10,13-dimethylhexadecahydro-1H-cyclopenta[a]phenan-thren-3-yl)oxy)-3,4,5-trihydroxytetrahy-dro-2H-pyran-2-carboxylic acid	C_29_H_46_O_10_	577.2983 [M+Na]^+^	577.2993	+1.8	5.925	Steroid glucuronide
**17**	Timosaponin A3	C_39_H_64_O_13_	763.4200 [M+Na]^+^	763.4204	+0.5	6.965	Steroidal saponin
**18**	(2*S*,3*S*,4*S*,5*R*,6*R*)-6-(((3*R*,5*R*,7*R*,8*R*,9*S*,10*S*,12*S*,13*R*,14*S*,17*R*)-17-((*R*)-4-carboxybutan-2-yl)-7,12-dihydroxy-10,13-dimethylhexadecahy-dro-1H-cyclopenta[a]phenan-thren-3-yl)oxy)-3,4,5-trihydroxytetrahy-dro-2H-pyran-2-carboxylic acid	C_30_H_48_O_11_	567.3164 [M-H_2_O+H]^+^	567.3160	−0.6	6.999	Steroid glucuronide

Note: Compounds marked with asterisk (*) contain prenyl (isoprenoid) substituents, representing meroterpenoid-type structures. Mass error (ppm) = [(Experimental *m*/*z* − Theoretical *m*/*z*)/Theoretical *m*/*z*] × 10^6^. Analytes with mass errors greater than 5 ppm (specifically Peaks **3**, **8**, **9**, **10**, **12**, and **13**) are considered tentative assignments based on accurate mass alone and warrant further confirmation by MS/MS fragmentation or authentic standards in future studies.

## Data Availability

The original contributions presented in this study are included in the article/Supplementary Materials. Further inquiries can be directed to the corresponding authors.

## Supplementary Materials

The following supporting information can be downloaded at: https://www.mdpi.com/article/10.3390/jof12080601/s1, Table S1: Composition of medium for seed culture and production culture (pH = 7).
