# Supplementary material for: Isolation, Identification, and Antimicrobial Activity of Secondary Metabolites from Pseudophaeolus soloniensis Against Phytopathogenic Fungi
Source: J Fungi (Basel). 2026 Aug 12;12(8):601. doi: 10.3390/jof12080601 (PMC13514609; doi:10.3390/jof12080601)
Supplement: Supplementary file 1 [file jof-12-00601-s001.zip › jof-4433992-supplementary.pdf]

Supporting Information

Isolation, Identification, and Antimicrobial Activity of Secondary Metabolites from *Pseudophaeolus soloniensis* Against Phytopathogenic Fungi

Chang Liu [1,2,3,*], Xiaoxue Zhao [1,2], Heng Zhao [1,2], Jun Zuo [1,2], Ruihan Wang [1], Mengshi Wang [2,3], Tianqi Wang [1,2], Jingyu Huang [1,2], Bin Du [2,3] and Kui Niu [1,2,3,*]

[1] College of Chemical Engineering, Hebei Normal University of Science and Technology, Qinhuangdao 066004, China; zxx4297@126.com (X.Z.); 18230248602@163.com (H.Z.); 15350674431@163.com (J.Z.); ruihwang@163.com (R.W.); 18232665658@163.com (T.W.); hzjy3657201@163.com (J.H.)

[2] Hebei Key Laboratory of Natural Products Activity Components and Function, Hebei Normal University of Science and Technology, Qinhuangdao 066004, China; wms15233551083@163.com (M.W.); bindufood@aliyun.com (B.D.)

[3] Engineering Research Centre of Chestnut Industry Technology Ministry of Education, Hebei Normal University of Science and Technology, Qinhuangdao 066004, China

* Correspondence: 15204042879@163.com (C.L.); niukui@hevttc.edu.cn (K.N.)

Table of Contents

Fermentation Optimization and Crude Extract Preparation **S1**

Fermentation Optimization and Crude Extract Preparation

Firstly, a loopful of strain culture from an agar plate was inoculated into a 500 mL flask containing 100 mL of V22 medium as the seed medium to increase the cell biomass. The flask was incubated on a rotary shaker (200 rpm) at 28 °C for approximately 1 week. Subsequently, 5 mL of the seed culture was inoculated into separate 500 mL flasks, each containing 100 mL of semi-solid A3M, A11M, and A16 production medium, respectively. The composition of these seed and production media is shown in Table S1. The pH of the media was adjusted to 7.0 prior to sterilization. The inoculated flasks were incubated at 28 °C in the dark for 7 days. The results showed that the fungus grew well only on the A3M solid medium. Therefore, large-scale solid-state fermentation was carried out on A3M solid medium in Petri dishes and maintained at 28 °C in the dark for 35 days. Finally, 1-butanol was selected as an ideal solvent for the extraction of all secondary metabolites from the fermentation medium due to its wide solubility range covering both hydrophilic and hydrophobic compounds. The combined organic layers were concentrated under reduced pressure at 40°C to obtain a brown, viscous crude extract. After weighing and designation as P1, the crude extract was stored at 4 °C until further analysis.

Table S1. Composition of medium for seed culture and production culture (pH = 7)

Composition (g/100 mL)	V22	A3M-Semisolid	A3M	A11M-Semisolid	A11M	A16-Semisolid	A16
Soluble starch	1.0	2.0	2.0		2.5	-	-
Glucose	0.5	0.5	0.5		0.2	2.0	2.0
NZ-amine	-	-	-		0.5	-	-
NZ-case	0.3	-	-		-	-	-
Glycerol	-	2.0	2.0		-	-	-
Yeast extract	0.2	0.3	0.3		0.5	-	-
Polypeptone	-	-	-		0.5	-	-
Tryptone	0.5	-	-		-	-	-
Pharmamedia	-	1.5	1.5		-	1.0	1.0
K_2HPO_4	0.1	-	-		-	-	-
$MgSO_4 \cdot 7H_2O$	0.04	-	-		-	-	-
$CaCO_3$	0.3	-	-		0.3	0.5	0.5
HP-20	-	1.0	1.0		1.0	1.0	1.0
Agar	1.0	0.6	1.0	0.6	1.0	0.6	1.0
